# POSTERIOR CRUCIATE LIGAMENT RECONSTRUCTION: ARE THE RESULTS SIMILAR TO ANTERIOR CRUCIATE LIGAMENT RECONSTRUCTION?

**DOI:** 10.1590/1413-785220233102e260740

**Published:** 2023-05-01

**Authors:** MARCOS BARBIERI MESTRINER, FÁBIO EIDI HIROSSE, NAYRA DEISE DOS ANJOS RABELO, ALFREDO DOS SANTOS NETTO, VICTOR MARQUES DE OLIVEIRA, RICARDO DE PAULA LEITE CURY

**Affiliations:** 1 Santa Casa de Misericórdia de Sao Paulo, Departamento de Ortopedia e Traumatologia, Grupo de Joelho, Sao Paulo, SP, Brazil.; 2 Santa Casa de Misericórdia de Sao Paulo, Departamento de Ortopedia e Traumatologia, Sao Paulo, SP, Brazil.; 3 Universidade Nove de Julho, Departamento de Ciências da Reabilitação, Laboratório de Análise do Movimento Humano, Sao Paulo, SP, Brazil.; 4 Santa Casa de Misericórdia de Sao Paulo, Faculdade de Ciências Médicas, Sao Paulo, SP, Brazil.

**Keywords:** Posterior Cruciate Ligament, Posterior Cruciate Ligament Reconstruction, Anterior Cruciate Ligament, Anterior Cruciate Ligament Reconstruction, Knee, Ligamento Cruzado Posterior, Reconstrução do Ligamento Cruzado Posterior, Ligamento Cruzado Anterior, Reconstrução do Ligamento Cruzado Anterior, Joelho

## Abstract

**Objective:**

To report and compare the results of posterior cruciate ligament (PCL) and anterior cruciate ligament (ACL) reconstructions.

**Methods:**

In total, 42 patients were retrospectively evaluated, 20 with isolated PCL injuries (group 1) and 22 with isolated ACL ones (group 2) who were subjected to arthroscopic ligament reconstruction with autologous grafts and followed up for at least two years. To evaluate the results in group 1, objective IKDC and Lysholm scores, posterior drawer tests, and evaluations by a KT-1000 arthrometer were used, whereas for group 2, subjective IKDC and Lysholm score and the Lachman test were employed. To compare groups, objective IKDC and Lysholm scores and assessment via a KT-1000 arthrometer were considered.

**Results:**

Intragroup analysis showed improved results for all variables (p < 0.001) in both groups. Comparisons between groups showed a significant difference in objective IKDC scores (p < 0.001), but no such disparities for Lysholm ones (p = 0.052), clinical tests (p = 0.058) or evaluation by KT-1000 (p = 0.129).

**Conclusion:**

Treatment restored knee stability and function in both groups. Comparisons between groups showed that PCL reconstructions had inferior results than ACL ones according to patients’ objective IKDC scores. ***Level of Evidence II, Retrospective Study.***

## RESUMO

**Objetivo:**

Reportar e comparar os resultados da reconstrução do ligamento cruzado posterior (LCP) e do ligamento cruzado anterior (LCA).

**Métodos:**

Foram avaliados retrospectivamente 42 pacientes: 20 com lesão isolada do LCP (grupo 1) e 22 com lesão isolada do LCA (grupo 2), submetidos à reconstrução ligamentar artroscópica com enxertos autólogos e acompanhados por pelo menos dois anos. Para avaliação dos resultados no grupo 1, foram utilizados o escore do International Knee Documentation Committee (IKDC) objetivo, escore de Lysholm, teste da gaveta posterior e avaliação pelo artrômetro KT-1000; e, para o grupo 2, foram utilizados o IKDC subjetivo, escore Lysholm e teste de Lachman. Para comparação entre os grupos, foram considerados o IKDC objetivo, escore Lysholm e avaliação pelo artrômetro KT-1000.

**Resultados:**

Ambos os grupos demonstraram melhora dos resultados na análise intragrupo em todas as variáveis (p < 0,001). Na comparação intergrupos, observou-se diferença significativa no IKDC objetivo (p < 0,001), não sendo observada diferença no escore Lysholm (p = 0,052), nos testes clínicos (p = 0,058) ou na avaliação pelo KT-1000 (p = 0,129).

**Conclusão:**

A estabilidade e função do joelho foram restauradas em ambos os grupos. Na comparação intergrupos, a reconstrução do LCP apresentou resultados inferiores à reconstrução do LCA no critério IKDC objetivo. **Nível de Evidência II, Estudo Retrospectivo.**

## INTRODUCTION

Most posterior cruciate ligament (PCL) injuries are composite and generate significant instability, functional impotence, and future degenerative changes. Research has well established the indication of surgery for this group of patients.^1^

Currently, doubts in the literature relate more to the much less frequent isolated PCL injuries, usually indicating surgical treatment for cases of grade III lesions, which, even if isolated, can cause disabling outcomes such as instability and anterior pain. Grade II chronic injuries can bring the same symptoms in young and active patients if conservative treatment fails. ^(^ ^1^ However, as PCL reconstruction techniques have evolved, including the advent of double-bundle reconstruction and better fixation methods, graft options, and rehabilitation protocols, the surgical treatment of isolated injuries has vastly improved, and the literature often reports superior results to those of conservative treatment. ^(^ ^2^ ^)- (^ ^4^

PCL reconstruction generally shows inferior results than those for anterior cruciate ligament (ACL). ^(^ ^5^ ^), (^ ^6^ However, via the aforementioned surgical advances, in 2018, LaPrade et al., ^(^ ^7^ ^)^ performed double-bundle PCL reconstructions with homologous grafts, obtaining comparable results for their isolated reconstruction of both ligaments.

Homologous grafts (or allografts) have been the preference for reconstructing PCL, especially for combined injures due to their shorter procedure time, lower surgical morbidity, and possibility of obtaining longer and more robust grafts. ^(^ ^1^ ^), (^ ^8^ ^), (^ ^9^ However, the Brazilian reality does not allow their wide use, often leaving autologous grafts as the only available option. Nevertheless, studies have successfully reconstructed PCL with autografts, often obtaining comparable results to allografts. ^(^ ^10^ ^)- (^ ^12^

We found only one study has compared the results of PCL and ACL reconstruction with autografts, ^(^ ^6^ unlike LaPrade et al., ^(^ ^7^ who used allografts. This study employed a simple bundle technique in most cases. Thus, as far as we know, this is the first study comparing the results of PCL and ACL reconstructions with autografts associated with double-bundle PCL reconstruction. We hypothesized that both can effectively restore knee stability and function and that their results would resemble each other even if we used autografts to reconstruct PCL.

## METHODS

This retrospective longitudinal study was approved by the research ethics committee of our institution (CAAE 40713720.1.0000.5479). From April 2002 to January 2017, results from two groups were reviewed: from patients who had been subjected to isolated double-bundle PCL reconstruction and those subjected to isolated ACL reconstruction who were followed up in our institution. All interventions were performed by a single surgeon (RPLC).

Skeletally mature patients of all genders with isolated and complete central ligament (PCL or ACL) injuries without associated ligament lesions who underwent outpatient follow-up for at least two years were included. Patients with clinical and/or radiographic signs of gonarthrosis, poor lower limb alignment, history of previous knee injuries or surgeries or who failed to follow our rehabilitation protocol were excluded.

From April 2002 to January 2017, 172 PCL injury cases were treated in our service, including combined or isolated injuries and cases of fracture-avulsion. From the criteria above, 20 patients were considered eligible for this study (group 1). Given that number, a random sample of 22 patients with isolated ACL injuries who were treated in the same period were selected (group 2).

### Outcome diagnosis and evaluation

Patients’ physical condition was thoroughly examined by two experienced orthopedists to diagnose ligament injuries. The anterior and posterior drawer, Lachman, Godfrey, pivot-shift, reverse pivot-shift, dial, varus and valgus opening (both at 0 and 30°), and external rotation recurvatum tests were performed.

Imaging tests were used to confirm patients’ diagnosis: radiography (frontal, profile, and panoramic images of participants’ lower limbs) - to evaluate their mechanical axes and the incidence of gonarthrosis and magnetic resonance imaging to assess ligament, chondral and/or meniscal lesions.

The following criteria were considered to assess stability and functionality preoperatively and postoperatively (within two years of follow-up) in group 1 (PCL): Lysholm and objective IKDC scores; posterior drawer test; and evaluation via a KT-1000 arthrometer; whereas for group 2 (ACL), Lysholm and subjective IKDC scores and the Lachman test.

To postoperatively compare groups, the available data common to both were considered and the following postoperative variables, compared after at least two years of follow-up: Lysholm and objective IKDC scores and evaluation via a KT-1000 arthrometer.

### Surgical technique

All cases included were operated by the same surgeon (R.P.L.C.). The transtibial technique in femoral double bundle^11^ ^)- (^ ^13^ with autologous quadriceps and semitendinosus grafts from patients’ ipsilateral knees or bilateral flexor tendons were used in PCL reconstructions (group 1), whereas the “outside-in” ^(^ ^14^ technique with autografts from ipsilateral flexor tendons was used in ACL reconstructions (group 2). If grafts only included flexor tendons, anterolateral bundles were reconstructed with two semitendinosus grafts and posteromedial ones with two gracilis ones. In cases treated with quadriceps and semitendinosus tendons, the former were used to reconstruct anterolateral bundles and the latter, posteromedial ones.

The anterolateral bundles in group 1 showed a 9-mm mean diameter in reconstructions with quadriceps tendons and a 8-mm mean diameter in those with double semitendinosus grafts. Posteromedial bundles treated with two gracilis grafts or a single semitendinosus ones showed a 7-mm average diameter. In group 2, both flexor tendons showed a 8-mm mean diameter.

Meniscal lesions were treated by partial meniscectomy and chondral lesions, by microfractures. Rehabilitation was standardized according to previous protocols. ^(^ ^14^ ^), (^ ^15^

A 5% statistical significance was defined and confidence intervals, constituted with 95% statistical confidence. The chi-squared test was used to compare the evaluated parameters within groups and between them.

## RESULTS

Table 1 shows both groups’ demographic distribution and associated lesions.

**Table 1:** Sample characterization.

Group	Gender		Age (average)
	M	F	
1 (PCL)	17 (85%)	3 (15%)	18-44 (32.64)
2 (ACL)	11 (50%)	11 (50%)	15-43 30.15

PCL: posterior cruciate ligament; ACL: anterior cruciate ligament; M: male; F: Female.

Tables 2 and 3 show the results of each group separately (intragroup evaluation). We found that all variables showed significant statistical evolution when we compared patients before and after surgery.

**Table 2:** Intragroup comparison of variables in group 1 - Posterior cruciate ligament reconstruction.

Outcomes N		Preop.		Post-p		p-value
		%	N	%		
**Lysholm**	**Poor**	7	35.0%	0	0.0%	< 0.001
	**Average**	9	45.0%	0	0.0%	
	**Good**	4	20.0%	8	40.0%	
	**Excellent**	0	0.0%	12	60.0%	
**Objective IKDC**	**D**	12	60.0%	0	0.0%	< 0.001
	**C**	8	40.0%	1	5.0%	
	**B**	0	0.0%	15	75.0%	
	**A**	0	0.0%	4	20.0%	
**Posterior drawer test**	**3+**	14	70.0%	0	0.0%	< 0.001
	**2+**	6	30.0%	1	5.0%	
	**1+**	0	0.0%	7	35.0%	
	**Negative**	0	0.0%	12	60.0%	
**KT-1000**	**> 10**	15	75.0%	0	0.0%	< 0.001
	**6 to 10**	5	25.%	1	5.0%	
	**3 to 5**	0	0.0%	7	35.0%	
	**0 to 2**	0	0.0%	12	60.0%	

PCL: posterior cruciate ligament; IKDC: International Knee Documentation Committee score; Preop: preoperative; Postop: postoperative; A: normal; B: almost normal; C: abnormal; D: Very abnormal.

**Table 3:** Intragroup comparison of variables in group 2 - Anterior cruciate ligament reconstruction.

Outcomes N		Preop.		Postop		p-value
		%	N	%	N	
**Lysholm**	**Poor**	12	54.5%	0	0.0%	< 0.001
	**Average**	7	31.8%	0	0.0%	
	**Good**	3	13.6%	3	13.6%	
	**Excellent**	0	0.0%	19	86.4%	
**Subjective IKDC**	**Poor**	19	86.4%	0	0.0%	< 0.001
	**Average**	3	13.6%	0	0.0%	
	**Good**	0	0.0%	5	22.7%	
	**Excellent**	0	0.0%	17	77.3%	
**Lachman Test**	**3+**	4	18.2%	0	0.0%	< 0.001
	**2+**	9	40,9%	0	0.0%	
	**1+**	9	40.9%	2	9.1%	
	**Negative**	0	0.0%	20	90.9%	

ACL: anterior cruciate ligament; IKDC: International Knee Documentation Committee score; Preop: preoperative; Postop: postoperative.

Table 4 shows our comparison of postoperative outcomes between groups (intergroup evaluation) for the variables which enabled analysis (Lysholm, objective IKDC, and evaluation by KT-1000). We only found a significant difference in objective IKDC scores (four patients classified as “A” in group 1 (20%) vs 19 in group 2 (86.4%) - p < 0.001). The Lysholm score failed to show statistical significance (p = 0.052), despite considerable numerical differences among patients deemed “excellent.”

**Table 4:** Intergroup comparison of postoperative variables.

Outcomes		Group 1 (PCL)		Group 2 (ACL)		p-value
		N	%	N	%	
**Lysholm**	**Poor**	0	0.0%	0	0.0%	0.052
	**Average**	0	0.0%	0	0.0%	
	**Good**	8	40.0%	3	13.6%	
	**Excellent**	12	60.0%	19	86.4%	
**Objective IKDC**	**D**	0	0.0%	0	0.0%	< 0.001
	**C**	4	5.0%	0	0.0%	
	**B**	15	75.0%	3	13.6%	
	**A**	4	20.0%	19	86.4%	
**Clinical trials (Lachman/PD)**	**3+**	0	0.0%	0	0.0%	0.058
	**2+**	1	5.0%	0	0.0%	
	**1+**	7	35.0%	2	9.1%	
	**Negative**	12	60.0%	20	90.9%	
**KT-1000**	**> 10 mm**	0	0.0%	0	0.0%	0.129
	**6 to 10 mm**	1	5.0%	0	0.0%	
	**3 to 5 mm**	7	35.0%	3	13.6%	
	**0 to 2 mm**	12	60.0%	19	86.4%	

PCL: posterior cruciate ligament; ACL: anterior cruciate ligament; PD: posterior drawer test; IKDC: International Knee Documentation Committee score; A: normal; B: almost normal; C: abnormal; D: very abnormal.

Finally, we found three cases of stiffness in group 1 (15%). Patients received additional manipulation under narcosis. We observed no complications in group 2.

## DISCUSSION

This study shows that the employed techniques were efficient, showing a high success rate in subjective and objective criteria after we compared patients before and after surgery. Nevertheless, postoperative intergroup comparisons showed that 86.4% of group 2 participants had “normal” operated knees, whereas only 20% of group 1 participants did. The other two variables showed no such situation, but we found a considerable difference in absolute Lysholm scores (p = 0.052, 86.4% and 60.0% “excellent” patients in group 2 and group 1, respectively).

Currently, the literature shows consensus on the need to surgically treat cruciate ligament injuries to reestablish knee stability and biomechanics and avoid secondary injuries and long-term joint degeneration. ^(^ ^16^ ^), (^ ^17^ ^), (^ ^18^ Thus, ligament reconstruction techniques have been improved, especially for PCL, showing better subjective and objective results. ^(^ ^1^ ^), (^ ^3^ However, PCL reconstruction results remain, in general, incomparable to those for ACL. ^(^ ^5^ ^), (^ ^6^

Our results disagree with LaPrade et al., ^(^ ^7^ ^)^ who showed a clear similarity between their isolated ACL and PCL reconstruction results. However, some important differences must be considered, the first of which includes grafts. LaPrade et al. ^(^ ^7^ used allografts (calcaneal tendons with bone blocks associated with anterior tibial tendons) to reconstruct PCL (including due to isolated injuries), whereas we used autografts, which demand a longer surgical time as they require surgical removal and show higher morbidity during collection. We believe that this may have interfered with our postoperative functional results.

Moreover, LaPrade et al. ^(^ ^7^ used a different graft fixation method. Although they also employed femoral interference screws, they performed tibial fixation by a screw with a toothed washer directly on the graft^7^. Allografts are generally longer than autografts, enabling direct fixation to the anterior portion of patients’ tibiae.

We inserted interference screws into tibial tunnels in 20% of our cases (those in which grafts consisted only of flexor tendons). In the remaining 80%, we employed a “on-post” fixation technique: we tied and tensioned the wires attached to the graft around a conventional screw and washer we had inserted more distally, creating a double interface - “screw-wires” and “graft-wires” - instead of more proximally and directly fixating the graft on the tibial anterior portion. This technique was necessary as quadriceps tendons produce shorter grafts which prohibit direct fixation. The “direct” method in LaPrade et al. may produce more stable and resistant fixations than the “post” fixation of autologous quadriceps tendon grafts, especially considering the great mechanical stress exerted on PCL reconstruction. ^(^ ^19^ A biomechanical study has shown that proximal fixation in patients’ tibial tunnels with or without distal fixation generated more stable results than the latter alone. ^(^ ^20^

Outcome evaluation methods also differed between studies: LaPrade et al. ^(^ ^7^ used SF-12, WOMAC, Tegner, and Lysholm scores. We only included the latter, and both studies showed no statistical difference. We found that the objective IKDC score was the only method showing a clear difference between our experimental groups, which LaPrade et al. ignored.

Differences in rehabilitation must also be considered. PCL cases require even more careful and intensive rehabilitation than ACL ones. ^(^ ^15^ In this study, although all patients underwent rehabilitation in the same institution under a standardized protocol, we must consider the difficulties of intensive follow-up due to socioeconomic limitations and the local public health system. Moreover, LaPrade et al. ^(^ ^7^ report routine dynamic orthosis in all patients who underwent PCL reconstruction, absent in Brazil due to economic limitations. This may also explain our treatment of patients’ associated injuries (meniscal and/or chondral) as the Brazilian public health system lacks the more advanced techniques to repair chondral lesions and suture menisci.

Finally, our results evade comparison with those in Owensen et al., ^(^ ^6^ who, despite their similar goal and use of autografts in most cases, only employed KOOS subjective scores to compare PCL and ACL reconstructions. They concluded that, although both groups show evident and comparable subjective improvements, patients subjected to PCL reconstruction show lower preoperative and postoperative KOOS scores.

This study has limitations. Its retrospective nature and convenience sample limit the interpretation of the effects of its interventions, especially considering its relatively small sample due to the infrequency of isolated PCL injuries. Moreover, we compared distinct lesions treated with different surgical techniques to evaluate similarities between the evolution of the two surgically treated injuries. We know that lesions and associated procedures can generate bias in result assessments, but it would be extremely difficult to obtain a “pure” sample - i.e., without any associated meniscal or chondral lesions - of an injury as rare as isolated PCL ones. To minimize this bias - exclusive to group 1 (PCL) -, we chose to pair both set of patients to avoid excluding those with associated meniscal injuries from group 2 (ACL), thus randomly distributing this possible bias across groups.

## CONCLUSION

Both PCL and LCA reconstructions managed to restore knee stability and function, showing significant improvement in our intragroup comparison. However, PCL reconstruction with autografts showed poorer results in objective IKDC scores than those for ACL. Intergroup analysis showed no statistically significant difference for our other variables.

## References

[1] Pache S, Aman ZS, Kennedy M, Nakama GY, Moatsche G, Ziegler C, LaPrade RF (2018). Posterior cruciate ligament current concepts review. Arch Bone Jt Surg.

[2] Kim YM, Lee CA, Matava MJ (2011). Clinical results of arthroscopic single-bundle transtibial posterior cruciate ligament reconstruction a systematic review. Am J Sports Med.

[3] Chahla J, Moatshe G, Cinque ME, Dornan GJ, Mitchell JJ, Ridley TJ, LaPrade RF (2017). Single-bundle and double-bundle posterior cruciate ligament reconstructions a systematic review and meta-analysis of 441 patients at a minimum 2 years&apos; follow-up. Arthroscopy.

[4] LaPrade CM, Civitarese DM, Rasmussen MT, LaPrade RF (2015). Emerging updates on the posterior cruciate ligament a review of the current literature. Am J Sports Med.

[5] Devitt BM, Dissanayake R, Clair J, Napier RJ, Porter TJ, Feller JA, Webster KE (2018). Isolated posterior cruciate reconstruction results in improved functional outcome but low rates of return to preinjury level of sport a systematic review and meta-analysis. Orthop J Sports Med.

[6] Owesen C, Sivertsen EA, Engebretsen L, Granan LP, Årøen A (2015). Patients with isolated PCL injuries improve from surgery as much as patients with ACL injuries after 2 years. Orthop J Sport Med.

[7] LaPrade RF, Cinque ME, Dornan GJ, DePhillipo NN, Geeslin AG, Moatsche G, Chahla J (2018). Double-bundle posterior cruciate ligament reconstruction in 100 patients at a mean 3 years’ follow-up outcomes were comparable to anterior cruciate ligament reconstructions. Am J Sports Med.

[8] Ansari AS, Dennis BB, Horner NS, Zhu M, Brookes C, Khan M, Grant JA (2019). Influence of graft source on postoperative activity and joint laxity in posterior cruciate ligament reconstruction: a systematic review. Arthroscopy.

[9] Belk JW, Kraeutler MJ, Purcell JM, McCarty EC (2018). Autograft versus allograft for posterior cruciate ligament reconstruction an updated systematic review and meta-analysis. Am J Sports Med.

[10] Mestriner MB, Cury RPL, Santos A, Oliveira VM, Camargo OPA, Belloti JC (2020). Double-bundle posterior cruciate ligament reconstruction no differences between two types of autografts in isolated or combined lesions. Knee.

[11] Cury RPL, Castro RN, Sadatsune DA, Prado DR, Gonçalves RJP, Mestriner MB (2017). Double-bundle PCL reconstruction using autologous hamstring tendons outcome with a minimum 2-year follow-up. Rev Bras Ortop.

[12] Cury RPL, Mestriner MB, Kaleka CC, Severino NR, Oliveira VM, Camargo OPA (2014). Double-bundle PCL reconstruction using autogenous quadriceps tendon and semitendinosus graft surgical technique with 2-year follow-up clinical results. Knee.

[13] Cury RPL, Severino NR, Camargo OPA, Aihara T, Oliveira VM, Avakian R (2012). Posterior cruciate ligament reconstruction with with autograft of the double semitendinosus muscles and middle third of the quadriceps tendon with double femoral and single tibial tunnels clinical results in two years follow up. Rev Bras Ortop.

[14] Cury RPL, Sprey JWC, Bragatto ALL, Mansano MV, Moscovici HF, Guglielmetti LGB (2017). Comparative evaluation of the results of three techniques in the reconstruction of the anterior cruciate ligament, with a minimum follow-up of two years. Rev Bras Ortop.

[15] Cury RPL, Kiyomoto HD, Rosal GF, Bryk FF, Oliveira VM, Camargo OPA (2012). Rehabilitation protocol after isolated posterior cruciate ligament reconstruction. Rev Bras Ortop.

[16] Shelbourne KD, Davis TJ, Patel DV (1999). The natural history of acute, isolated, nonoperatively treated posterior cruciate ligament injuries A prospective study. Am J Sports Med.

[17] Boynton MD, Tietjens BR (1996). Long-term followup of the untreated isolated posterior cruciate ligament-deficient knee. Am J Sports Med.

[18] Filbay SR, Grindem H (2019). Evidence-based recommendations for the management of anterior cruciate ligament (ACL) rupture. Best Pract Res Clin Rheumatol.

[19] Wijdicks CA, Kennedy NI, Goldsmith MT, Devitt BM, Michalski MP, Årøen A (2013). Kinematic analysis of the posterior cruciate ligament, part 2 a comparison of anatomic single-versus double-bundle reconstruction. Am J Sports Med.

[20] Zhang X, Teng Y, Li R, Ma C, Yang X, Wang H (2019). Proximal, distal, and combined fixation within the tibial tunnel in transtibial posterior cruciate ligament reconstruction a time-zero biomechanical study in vitro. Arthroscopy.

